# Sublingual misoprostol and hyperpyrexia: case report with temperature curve

**DOI:** 10.1186/s13104-017-2661-2

**Published:** 2017-07-26

**Authors:** Paul Nkemtendong Tolefac, Jacqueline Ze Minkande

**Affiliations:** 1 Douala General Hospital, Douala, Cameroon; 2 Intern Faculty of Medicine and Biomedical Sciences, University of Yaoundé 1, Yaounde, Cameroon

**Keywords:** Misoprostol, Hyperpyrexia, Temperature, Case report

## Abstract

**Background:**

Misoprostol has a wide range of applications in obstetrics and gynaecology. It is widely recommended by WHO, FIGO and ACOG for the treatment of postpartum haemorrhage due to it safety and cost-effectiveness. However, usage might be associated to hyperpyrexia and shivering.

**Case presentation:**

We present a 30 year old Cameroonian female gravida 1 para 1 who had a vaginal delivery at 40 weeks of gestation complicated by primary postpartum haemorrhage (PPH). PPH was managed by sublingual misoprostol that induced shivering and hyperpyrexia managed successfully with paracetamol and cooling.

**Conclusions:**

The occurrence of fever and shivering should be kept in mind when administering misoprostol for PPH.

## Background

Postpartum haemorrhage (PPH) remains the most common cause of maternal mortality with uterine atony being the commonest underling aetiology [1, 2]. It accounts for nearly one-quarter of all maternal deaths worldwide and an estimated 125,000 deaths occur each year [1]. About 50% of these deaths occur in sub-Sahara Africa alone [3]. Researchers have for decades searched the safest, fastest and most effective pharmacological agents to manage this dreaded complication [4]. Conventional uterotonics such as oxytocin have been the drug of choice in the first line management of PPH secondary to uterine atony. However, a major short coming has been its obligate parenteral route of administration [2, 5].

Misoprostol is an attractive alternative because of its uterotonic potency, multiple convenient routes of administration and stability at ambient temperatures [6]. The most common side effects associated with the postpartum administration of misoprostol are shivering and pyrexia [7]. Studies show the rates of shivering and fever to be related to the dose and route of administration. Higher rates of shivering and elevated body temperature are associated with oral and sublingual routes of administration, which achieve a higher and faster maximum plasma concentration than vaginal and rectal routes [6–8]. We report the case of a 30 year old Cameroonian female gravida 1 para 1 who had a vaginal delivery at 40 weeks of gestation complicated by primary postpartum haemorrhage (PPH). PPH was managed by sublingual misoprostol that induced shivering and hyperpyrexia managed successfully with paracetamol and cooling.

## Case report

**Fig. 1:**
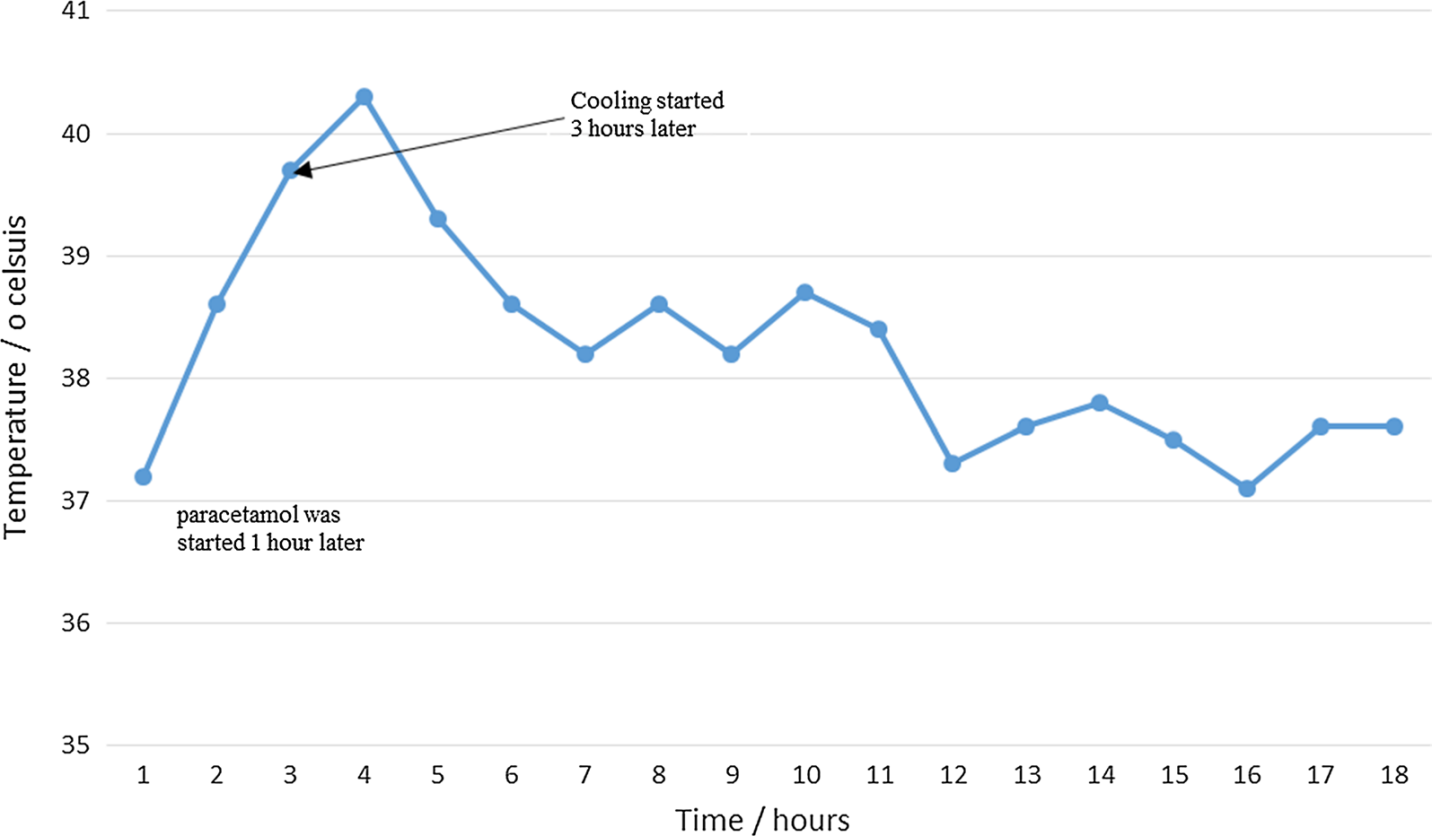
Temperature variation after sublingual misoprostol administration, paracetamol was started immediately and cooling by tepid sponging started 2 h later. The plot started 20 min after misoprostol administration. Paracetamol 1 g was administered 1 h after misoprostol administration and thereafter repeated every 6 h. Cooling was started 3 h after misoprostol administration

A 30 year old female Cameroonian gravida 1 para 1 at 40 weeks of gestation with an uneventful antenatal consultation presented at the active phase of labour with a cervical dilatation of 4 cm and good uterine contractions. Labour progressed normally 5 h later to the vaginal delivery of a live female baby with weight 2820 g and Apgar 8/10 and 10/10 at the first and 5th min respectively. Blood loss after delivery was estimated at 350 cc. Whilst active management of the third stage of labour was done with injection of 10 IU of oxytocin, the placenta was delivered, examined and found to be complete. At the end of the third stage of labour, the uterus was well contracted and globular. Patient was then transferred to the ward in a haemodynamically stable condition following 2 h of monitoring. Ten hours later and after evaluation she complained of abundant lochia associated with vertigo and physical examination revealed blood pressure 102/77, heart rate of 104 beats/min and temperature of 37.6 °C. The uterus was tender and poorly contracted, other aspects of the examination were normal. We concluded on mild uterine atony and placed 600 µg of misoprostol sublingual. 20 min later we were called for sudden shivering and cold sensation. The temperature measured was 37.2 °C, an hour later the temperature rose to 38.6 °C and two hours later to 39.7 °C (Fig. 1 showed temperature variations over 18 h). There was no dyspnoea, no itching, no rashes, no convulsion and no angioedema. Vital signs were stable and there was no cardiopulmonary distress. We concluded on a working diagnosis of hyperpyrexia from misoprostol. Emergency full blood count and C-reactive protein (CRP) were normal. The management consisted of paracetamol 1 g 6 hourly and cooling the patient via tepid sponging. Paracetamol and cooling were started 1 and 3 h respectively after misoprostol administration. Twelve hours later the temperature dropped and remained less than 38 °C and she was monitored for another 24 h and discharged.

## Discussion

Misoprostol, a synthetic prostaglandin E1 analogue originally used for the treatment of NSAID induced peptic ulcer has been found to have a wider application in the field of obstetrics and gynaecology because of its uterotonic and cervical-maturation effects [6]. It has been recommended for the treatment and prevention of PPH by ACOG, FIGO and WHO [9–11]. Compared to other uterotonics, misoprostol is cheap, readily available, has a longer shelf life, stable at ambient temperature, and can be administered easily. It is a very safe drug associated with transient, mild side-effects like fever, chills, nausea, vomiting, diarrhoea and abdominal pain [6]. Collective total daily doses of 1600 Âµg have been tolerated with only mild gastrointestinal discomfort [6]. A study done in Ecuador in 2010 showed that there was a sharp increase in temperature within 1 h of treatment, a peak in temperature 1–2 h post-treatment, and a gradual decline in temperature over a period of 3 h. Average temperatures remained above 40.0 °C for less than 2 h, and measured below 38.0 °C approximately 6 h after receiving misoprostol. In our indexed case shivering started 20 min after administration and the temperature started rising 1 h later reaching a peak of 40.3 °C 4 h later and dropping to less than 38.0 °C 12 h later. This was consistent with results of recent studies [4, 12]. Temperature elevations associated with the use of misoprostol are compatible with the hypothalamic adjustment. Prostaglandins E2 (PGE2) have been involved in the pathophysiological mechanism of endogenous fever and identified as the major mediator for inducing fever because of its interaction with the Prostaglandin E3 (PGE3) receptor. Misoprostol-induced fever mimics the PGE2 endogenous thermoregulation patterns, changing the hypothalamic adjustment in its upper segment and stimulating temperature elevation [4]. In our patient the possibility of a postpartum infection was almost zero as our patient had no risk factor such as prolong labour, premature rupture of membranes, etc. and emergency CRP and FBC had values within normal ranges. The fever is usually treated with paracetamol, anti-inflammatory and cooling [4, 12]. In our indexed case we used paracetamol and cooling and there was a good clinical response with temperature remaining less than 38 °C after 12 h (see Fig. 1).

## Conclusion

Hyperpyrexia associated with shivering may be associated with administration of misoprostol 600 µg, this is more common after sublingual administration. Treatment is with oral paracetamol and cooling. This common side effect should be kept in mind when administering misoprostol for postpartum haemorrhage.
